# Copper-catalyzed arylation of alkyl halides with arylaluminum reagents

**DOI:** 10.3762/bjoc.11.261

**Published:** 2015-12-02

**Authors:** Bijay Shrestha, Ramesh Giri

**Affiliations:** 1 Department of Chemistry & Chemical Biology, The University of New Mexico, Albuquerque, NM 87131, USA

**Keywords:** alkylation, arylalkanes, copper, cross-coupling, organoaluminum

## Abstract

We report a Cu-catalyzed coupling between triarylaluminum reagents and alkyl halides to form arylalkanes. The reaction proceeds in the presence of *N*,*N*,*N’*,*N’*-tetramethyl-*o*-phenylenediamine (NN-1) as a ligand in combination with CuI as a catalyst. This catalyst system enables the coupling of primary alkyl iodides and bromides with electron-neutral and electron-rich triarylaluminum reagents and affords the cross-coupled products in good to excellent yields.

## Introduction

Cross-coupling reactions represent one of the most important transformation for carbon–carbon (C–C) bond formation in organic synthesis [1–9]. These reactions, typically catalyzed by Pd and Ni, exploit a wide range of organometallic reagents of Mg, Zr, Zn, Sn, Al, B, Si and In as sources of nucleophiles. Among these metals/non-metals, Al offers a unique feature due to its high chemoselectivity and Lewis acidity [10–12]. In addition, Al also has low toxicity and is an inexpensive and earth-abundant metal. Organoaluminum reagents can be prepared directly from metallic aluminum [13–15], which further highlights the potential scope of these reagents in organic synthesis. However, despite extensive investigations and applications of organometallic reagents of Si, B, Mg, Zn and Sn in cross-coupling, the utility of organometallic complexes of Al are limited [13–14,16–21]. In many cases, direct transmetalation of organoalanes to Pd are sluggish and require ZnCl_2_ or CdCl_2_ to facilitate reactions through sequential transmetalations [19]. In some cases, intramolecular coordination to Al also enables the couplings of alkylalanes with organo halides [21]. Knochel [15] and Hoveyda [22] have also shown that organoaluminum reagents are capable of transmetalating to Cu-salts. Inspired by these literature reports and our recent investigations, we envisioned that organoaluminum reagents could participate as nucleophile sources in Cu-catalyzed cross-coupling reactions. In this artcle, we show that triarylaluminum reagents are excellent coupling partners for Cu-catalyzed cross-coupling reactions. The reaction proceeds for the coupling with primary alkyl iodides and bromides in good to excellent yields.

## Results and Discussion

**Scheme 1:**
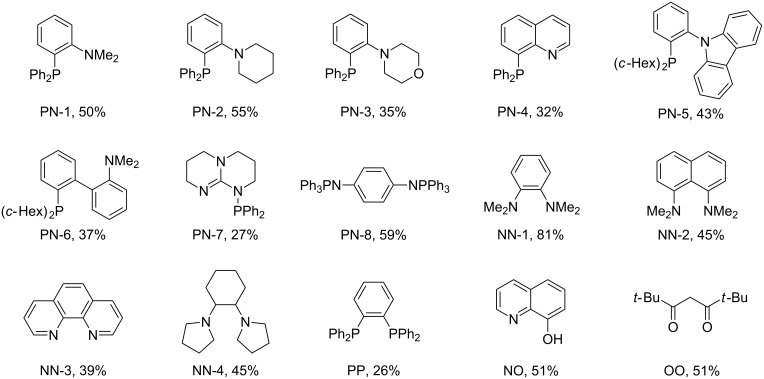
Ligands used for reaction optimization.

Recently, we [23–27] and others [10,28–39] reported efficient cross-couplings of oganometallic reagents of Si, B, In, Zr, Zn, Mg and Sn with organo halides [40–41]. Under our reaction conditions, a catalyst derived from the combination of CuI and 2-(diphenylphosphino)-*N*,*N*-dimethylaniline (PN-1) remains highly effective for coupling many of these organometallic reagents with aryl halides. In order to expand the scope of our coupling reactions, we utilized the standard condtions for the reaction of commercially available Ph_3_Al with 1-iodooctane using 1 mol % each of CuI and PN-1. However, the product, 1-phenyloctane (**3**), was formed only in 34% yield (Table 1, entry 1). Further optimization of the reaction conditions revealed that the coupling proceeded in 66% GC yield when the reaction was performed in NMP using 1 equivalent of Cs_2_CO_3_ as a base and 3 equivlents of LiCl as an additive in the absence of a ligand (Table 1, entry 2). We then screened a variety of ligands (Scheme 1) and found that *N*,*N*,*N’*,*N’*-tetramethyl-*o*-phenylenediamine (NN-1) was an efficient ligand for CuI that enabled us to increase the product yield to 81% GC yields (76% isolated, Table 1, entry 3) [10,35,42–44]. Reactions containing other PN- and NN-based ligands that are analogous to PN-1 and NN-1 (Scheme 1) afforded cross-coupled product **3** in lower yields than the reaction performed in the absence of NN-1. Reactions containing the bisphosphine ligand, *o*-bis(diphenylphosphine)benzene (PP) and anionic ligands such as 8-hydroxyquinoline (NO) and 2,2,6,6-tetramethyl-3,5-heptanedione (OO, Scheme 1) also formed the product **3** in lower yields and the reaction performed in the absence of NN-1. The reaction does not proceed in the absence of CuI (Table 1, entry 4). The cross-coupled product **3** is formed in 50% and 54% yield, respectively, in the absence of LiCl and Cs_2_CO_3_ (Table 1, entries 5 and 6). The reacton with 2 and 4 equivalents of LiCl also afforded product **3** in comparable yields (78% and 76%, respectively) to that of the standard reaction (Table 1, entries 7 and 8). However, excess of LiCl was found to be detrimental to the reaction (Table 1, entry 9). The reaction could also be performed at a temperature as low as 80 °C affording the coupled product **3** only in slightly lower yields than that of the standard reaction (Table 1, entries 10 and 11).

**Table 1:** Optimization of reaction conditions^a^.

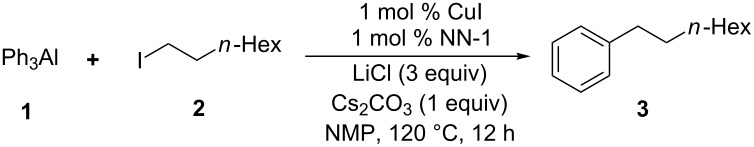		

Entry	Variation from the standard conditions	Yield (%)^b^

1	PN-1 instead of NN-1 in DMF, no Cs_2_CO_3_	34
2	No NN-1	66
3	none	81 (76)
4	without CuI	0
5	without LiCl	50
6	without Cs_2_CO_3_	54
7	2 equiv LiCl	78
8	4 equiv LiCl	76
9	6 equiv LiCl	35
10	100 °C	78
11	80 °C	75

^a^Reactions were run in 0.5 mL DMF. Commercially available Ph_3_Al was used. ^b^GC yields (average of at least two parallel runs) calibrated against 2-nitrobiphenyl as an internal standard. Value in parenthesis is the isolated yield (1.0 mmol).

After establishing the combination of NN-1 and CuI as the best catalyst, we began to explore the substrate scope of the reaction. While the reaction proceeded in good yields with alkyl iodides (Table 2, entries 1–3) by using 1 mol % of the catalyst, reactions with alkyl bromides, which are more readily available and less expensive than alkyl iodides, required 10 mol % of NN-1/CuI (Table 2, entries 4–15). The reaction can be performed with electron-neutral and electron-rich triarylaluminum reagents [45]. The reaction tolerates a variety of functional groups on alkyl halides including highly sensitive esters (Table 2, entries, 5, 9 and 11), nitriles (Table 2, entries 6 and 7) and olefins (Table 2, entries 4, 8, 10, 13 and 15) [46]. With 10 mol % catalyst loading, the reaction can also be extended to the coupling of triarylaluminum reagents with benzyl bromides (Table 2, entries 12 and 14) [43].

**Table 2:** Coupling of triarylaluminum reagents with alkyl iodides and bromides^a^.

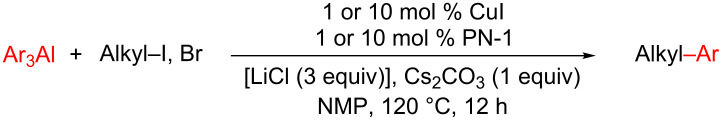				

Entry	Ar in Ar_3_Al	Alkyl−I,Br	Alkyl−Ar	yield (%)^b^

1	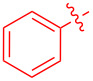	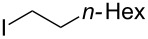	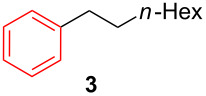	76
2	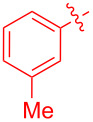	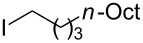	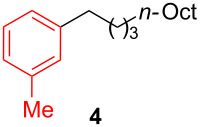	61
3	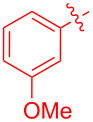	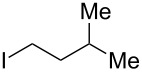	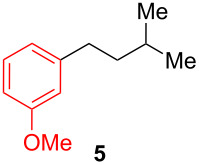	49
4	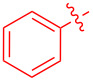	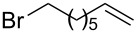	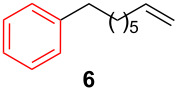	60
5	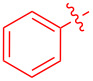	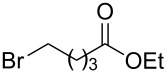	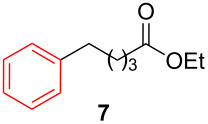	58
6	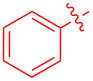	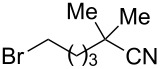	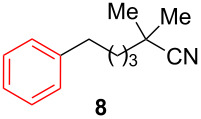	71
7	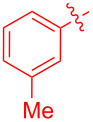	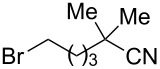	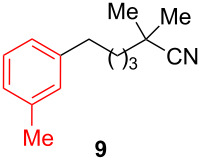	88
8	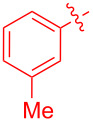	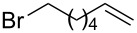	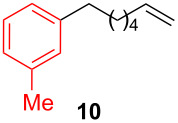	53
9	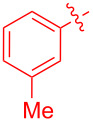	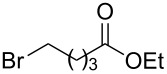	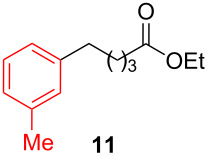	59
10	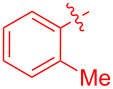	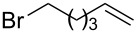	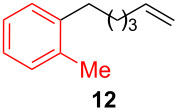	46
11	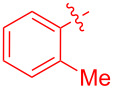	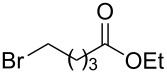	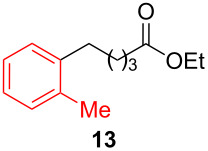	92
12	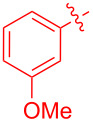	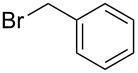	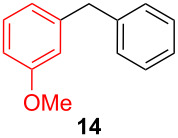	53
13	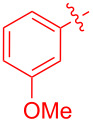	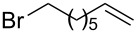	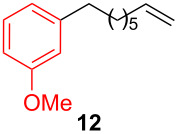	52
14	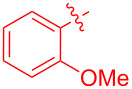	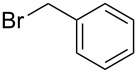	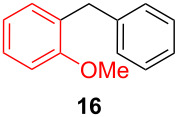	68
15	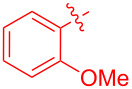	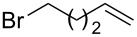	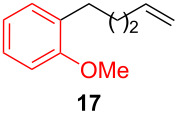	47

^a^Reactions were run in 5 mL DMF. Reactions for entries 1–3 were run with 1 mol % NN-1/CuI. Reactions for entries 4–15 were run with 10 mol % NN-1/CuI. Triarylaluminum reagents, except the commercially available Ph_3_Al, were prepared from the reaction of 3 equivalents of ArLi reagents with AlCl_3_ (99.99% purity) in THF at room temperature and were used without further purification. Each reaction contains 3 equivalents of LiCl, written in parenthesis below the reaction arrow, which is generated during the preparation of triarylaluminum reagents. ^b^Yields are for products isolated by column chromatography from a 1.0 mmol scale reaction.

**Scheme 2:**
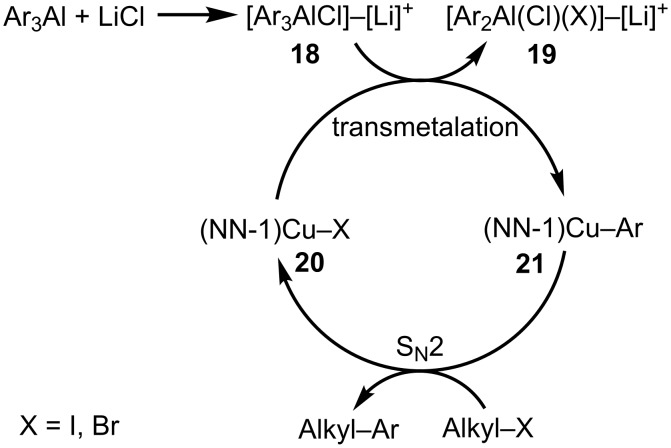
Proposed catalytic cycle.

Based on literature reports and our recent mechanistic work on Cu-catalyzed cross-couplings [23–25], we propose a catalytic cycle for the current reaction (Scheme 2). It is evident from the optimization of reaction conditions that both NN-1 and LiCl improve product yields for the current coupling of triarylaluminum reagents with alkyl halides (Table 1). As such, we believe that organoaluminate complexes such as **18**, generated from the binding of LiCl to three-coordinate triarylaluminum reagents, are the actual species in solution that undergo transmetalation with NN-bound CuX (X = I, Br) to generate (NN)CuAr complexes as the reaction intermediates. Catalytically competent Cu^I^-complexes that contain nitrogen-based ligands have previously been synthesized and fully characterized structurally [47–51]. Triorganoaluminum complexes are also known to form triorganoaluminate species in the presence of anions in solution [52–57]. In addition, organoaluminum reagents have been demonstrated to undergo transmetalation with Cu salts based on their participation in allylic and conjugate addition reactions [11,15,43]. Similar Cu-catalyzed couplings of organometallic reagents with alkyl electrophiles have previously been shown to proceed via an S_N_2 process [34–35]. Therefore, we believe that a similar mechanistic scenario can also be envisioned in the current Cu-catalyzed cross-coupling of triarylaluminum reagents with primary alkyl halides that involves (NN)CuAr as the reaction intermediates.

## Conclusion

In summary, we have developed a Cu-catalyzed coupling of triarylaluminum reagents with primary alkyl iodides and bromides. The reaction proceeds in the presence of NN-1/CuI as an effective catalyst. Electron-neutral and electron-rich triarylaluminum reagents can be coupled with a variety of alkyl iodides and bromides containing a range of sensitive functional groups such as olefins, nitriles and esters, affording the alkylated arenes in good to excellent yields.

## Experimental

**General information.** All the reactions and handling of chemicals were done inside a nitrogen-filled glovebox unless stated otherwise. All glassware were dried in an oven before use. All commercial reagents were used as received without further purification. Anhydrous solvents and triphenylaluminum were purchased from Sigma-Aldrich. Pure triarylaluminum reagents other than Ph_3_Al were synthesized following the reported procedure [56]. Ligands PN-5, PN-6, NN-2, NN-3, PP, NO and OO were purchased from commercial sources. Ligands PN-1, PN-2, PN-3, PN-4, PN-7 [58], PN-8, NN-1 [59], and NN-4 [60] were synthesized following the reported procedures [61]. ^1^H and ^13^C NMR spectra were recorded on a Bruker instrument (300 and 75 MHz, respectively) and internally referenced to the residual solvent signals of CDCl_3_ at 7.26 and at 77.16 ppm, respectively.

**General procedure for cross-coupling.** To a suspension of AlCl_3_ (133.3 mg, 1.0 mmol) in THF (2 mL) was added dropwise a solution of aryllithium (3.0 mmol, generated from the lithiation of aryl iodides with 1 equiv of *n*-BuLi in THF) at room temperature. After 45 minutes, the solvent was removed to obtain a triarylaluminum reagent containing 3 equivalents of LiCl, which was then dissolved in NMP (5 mL). Alkyl halide (1.0 mmol), CuI (1.9 mg, 0.010 mmol, for alkyl iodides; 19.0 mg, 0.10 mmol, for alkyl bromides) and NN-1 (1.6 mg, 0.010 mmol, for alkyl iodides; 16.4 mg, 0.10 mmol, for alkyl bromides) were then added to the solution of the triarylaluminum reagent. The reaction mixture was then tightly capped, taken out of the glovebox, placed in an oil bath pre-heated to 120 °C and vigorously stirred. After 12 h, the reaction mixture was cooled to room temperature, diluted with ethyl acetate (15 mL) and washed with H_2_O (5 mL × 3). The aqueous fraction was extracted back with ethyl acetate (5 mL × 3) and combined with the first ethyl acetate fraction. The combined ethyl acetate fractions were dried over Na_2_SO_4_ and the solvent was removed on a rotary evaporator. The product was purified by silica gel column chromatography using 0–5% ethyl acetate in hexanes.

***n*-Octylbenzene (3)** [62]: The title compound **3** was obtained as a colorless oil (144 mg, 76% yield) after purification by silica gel column chromatography. ^1^H NMR (300 MHz, CDCl_3_) δ 0.89 (t, *J* = 6.6 Hz, 3H), 1.29–1.32 (m, 10H), 1.58–1.68 (m, 2H), 2.62 (m, *J* = 8.1 Hz, 2H), 7.16–7.21 (m, 3H), 7.27–7.32 (m, 2H); ^13^C NMR (75 MHz, CDCl_3_) δ 14.2, 22.8, 29.4, 29.5, 29.6, 31.7, 32.0, 36.1, 125.7, 128.3, 128.5, 143.1; GC–MS (*m*/*z*) 190.1.

**1-Dodecyl-3-methylbenzene (4):** The title compound **4** was obtained as yellow oil (159 mg, 61% yield) after purification by silica gel column chromatography. ^1^H NMR (300 MHz, CDCl_3_) δ 0.90 (t, *J* = 6.3 Hz, 3H), 1.28–1.31 (m, 18H), 1.53–1.64 (m, 2H), 2.35 (s, 3H), 2.55–2.60 (m, 2H), 6.99–7.02 (m, 3H), 7.18 (t, *J* = 6.9 Hz, 1H); ^13^C NMR (75 MHz, CDCl_3_) δ 14.3, 21.6, 22.9, 27.1, 29.5, 29.6, 29.7, 29.8, 31.7, 32.1, 36.1, 45.3, 125.5, 126.4, 128.3, 129.4, 137.9, 143.1; GC–MS (*m*/*z*) 260.1.

**1-Isopentyl-3-methoxybenzene (5):** The title compound **5** was obtained as colorless oil (87 mg, 49% yield) after purification by silica gel column chromatography. ^1^H NMR (300 MHz, CDCl_3_) δ 0.93 (s, 6H), 1.46–1.64 (m, 3H), 2.6 (t, *J* = 7.8 Hz, 2H), 3.80 (s, 3H), 6.71–6.80 (m, 2H), 7.12–7.38 (m, 2H); ^13^C NMR (75 MHz, CDCl_3_) δ 22.7, 27.8, 33.9, 40.8, 55.2, 110.9, 114.3, 120.9, 129.3, 144.9, 159.7; GC–MS (*m*/*z*) 178.1.

**7-Octen-1-ylbenzene (6)** [63]: The title compound **6** was obtained as a colorless oil (113 mg, 60% yield) after purification by silica gel column chromatography. ^1^H NMR (300 MHz, CDCl_3_) δ 1.33–1.41 (m, 6H), 1.60–1.65 (m, 2H), 2.00–2.08 (m, 2H), 2.61 (t, *J* = 7.5 Hz, 2H), 4.91–5.03 (m, 2H), 5.75–5.88 (m, 1H), 7.17–7.20 (m, 3H), 7.25–7.31 (m, 2H); ^13^C NMR (75 MHz, CDCl_3_) δ 28.9, 29.1, 29.2, 31.5, 33.9, 36.0, 114.3, 125.6, 128.3, 128.5, 139.2, 142.9; GC–MS (*m*/*z*) 188.1.

**Ethyl 5-phenylvalerate (7)** [64]: The title compound **7** was obtained as yellow oil (120 mg, 58% yield) after purification by silica gel column chromatography. ^1^H NMR (300 MHz, CDCl_3_) δ 1.26 (t, *J* = 9.0 Hz, 3H), 1.66–1.70 (m, 4H), 2.31–2.35 (m, 2H), 2.62–2.67 (m, 2H), 4.13 (q, *J* = 6.9 Hz, 2H), 7.17–7.22 (m, 3H), 7.26–7.32 (m, 2H); ^13^C NMR (75 MHz, CDCl_3_) δ 14.3, 24.7, 33.5, 34.2, 35.6, 60.3, 125.8, 128.4, 128.4, 142.2, 173.7; GC–MS (*m*/*z*) 206.1.

**2,2-Dimethyl-6-phenylhexanenitrile (8)** [65]: The title compound **8** was obtained as yellow oil (143 mg, 71% yield) after purification by silica gel column chromatography. ^1^H NMR (300 MHz, CDCl_3_) δ 1.34 (s, 6H), 1.54–1.58 (m, 4H), 1.62–1.72 (m, 2H), 2.63–2.68 (m, 2H), 7.17–7.23 (m, 3H), 7.26–7.33 (m, 2H); ^13^C NMR (75 MHz, CDCl_3_) δ 25.0, 26.7, 31.5, 32.4, 35.7, 40.9, 125.2, 125.8, 128.4, 142.2; GC–MS (*m*/*z*) 201.1.

**2,2-Dimethyl-6-(3-methylphenyl)hexanenitrile (9):** The title compound **9** was obtained as yellow oil (189 mg, 88% yield) after purification by silica gel column chromatography. ^1^H NMR (300 MHz, CDCl_3_) δ 1.35 (s, 6H), 1.55–1.59 (m, 4H), 1.63–1.72 (m, 2H), 2.36 (s, 3H), 2.61–2.67 (m, 2H), 6.99–7.02 (m, 3H), 7.19 (t, *J* = 3.9 Hz, 1H); ^13^C NMR (75 MHz, CDCl_3_) δ 21.4, 25.0, 26.6, 31.5, 32.3, 35.6, 41.3, 125.2, 125.3, 126.5, 128.2, 129.2, 137.8, 142.1; GC–MS (*m*/*z*) 215.1.

**1-(6-Hepten-1-yl)-3-methylbenzene (10):** The title compound **10** was obtained as colorless oil (100 mg, 53% yield) after purification by silica gel column chromatography. ^1^H NMR (300 MHz, CDCl_3_) δ 1.34–1.45 (m, 4H), 1.59–1.66 (m, 2H), 2.02–2.09 (m, 2H), 2.33 (s, 3H), 2.57 (t, *J* = 7.8 Hz, 2H), 4.92–5.03 (m, 2H), 5.75–5.88 (m, 1H), 6.97–7.00 (m, 3H), 7.17 (t, *J* = 3.9 Hz, 1H); ^13^C NMR (75 MHz, CDCl_3_) δ 28.8, 29.7, 31.4, 33.7, 35.6, 114.2, 125.4, 126.3, 128.1, 129.2, 137.7, 139.1, 142.8; GC–MS (*m*/*z*) 188.2.

**Ethyl 5-(3-methylphenyl)valerate (11)** [66]: The title compound **11** was obtained as a yellow oil (130 mg, 59% yield) after purification by silica gel column chromatography. ^1^H NMR (300 MHz, CDCl_3_) δ 1.28 (t, *J* = 7.2 Hz, 3H), 1.66–1.72 (m, 4H), 2.33–2.37 (m, 5H), 2.60–2.65 (m, 2H), 4.15 (q, *J* = 7.2 Hz, 2H), 6.98–7.04 (m, 3H), 7.18 (t, *J* = 7.5 Hz, 1H); ^13^C NMR (75 MHz, CDCl_3_) δ 14.3, 21.5, 24.7, 31.0, 34.3, 35.6, 60.2, 125.4, 126.5, 128.3, 129.3, 137.9, 142.2, 173.7; GC–MS (*m*/*z*) 220.2.

**1-(5-Hexen-1-yl)-2-methylbenzene (12)** [67]: The title compound **12** was obtained as colorless oil (80 mg, 46% yield) after purification by silica gel column chromatography. ^1^H NMR (300 MHz, CDCl_3_) δ 1.48–1.67 (m, 4H), 2.09–2.16 (m, 2H), 2.33 (s, 3H), 2.59–2.65 (m, 2H), 4.98–5.07 (m, 2H), 5.77–5.91 (m, 1H), 7.11–7.15 (m, 4H); ^13^C NMR (75 MHz, CDCl_3_) δ 19.4, 29.1, 29.9, 33.3, 33.8, 114.6, 125.9, 125.9, 128.9, 130.2, 135.9, 139.0, 141.0; GC–MS (*m*/*z*) 174.1.

**Ethyl 5-(2-methylphenyl)valerate (13)** [68]: The title compound **13** was obtained as yellow oil (203 mg, 92% yield) after purification by silica gel column chromatography. ^1^H NMR (300 MHz, CDCl_3_) δ 1.26 (t, *J* = 7.2 Hz, 3H), 1.58–1.76 (m, 4H), 2.31–2.37 (m, 5H), 2.6–2.65 (m, 2H), 4.13 (q, *J* = 7.2 Hz, 2H), 7.11–7.12 (m, 4H); ^13^C NMR (75 MHz, CDCl_3_) δ 14.4, 19.4, 25.1, 29.8, 33.1, 34.4, 60.4, 126.0, 128.9, 130.3, 135.9, 140.5, 173.8; GC–MS (*m*/*z*) 220.2.

**1-Benzyl-3-methoxybenzene (14)** [69]: The title compound **14** was obtained as a light yellow oil (105 mg, 53% yield) after purification by silica gel column chromatography. ^1^H NMR (300 MHz, CDCl_3_) δ 2.82 (s, 3H), 3.8 (s, 2H), 6.77–6.84 (m, 2H), 6.91 (t, *J* = 9.0 Hz, 1H), 7.20–7.34 (m, 6H); ^13^C NMR (75 MHz, CDCl_3_) δ 42.0, 55.2, 111.4, 114.9, 121.4, 126.2, 128.3, 128.5, 129.5, 140.9, 142.7, 159.8; GC–MS (*m*/*z*) 198.1.

**1-Methoxy-3-(oct-7-en-1-yl)benzene (15):** The title compound **15** was obtained as colorless oil (113 mg, 52% yield) after purification by silica gel column chromatography. ^1^H NMR (300 MHz, CDCl_3_) δ 1.27–1.42 (m, 4H), 1.55–1.65 (m, 4H), 2.01–2.05 (m, 2H), 2.59 (t, *J* = 7.5 Hz, 2H), 3.81 (s, 3H), 4.92–5.02 (m, 2H), 5.75–5.88 (m, 1H), 6.72–7.36 (m, 4H); ^13^C NMR (75 MHz, CDCl_3_) δ 29.1, 29.3, 29.6, 31.4, 33.9, 35.8, 55.2, 110.9, 114.3, 121.0, 129.3, 129.8, 139.3, 144.7; GC–MS (*m*/*z*) 218.2.

**1-Benzyl-2-methoxybenzene (16)** [70]: The title compound **16** was obtained as light yellow oil (135 mg, 68% yield) after purification by silica gel column chromatography. ^1^H NMR (300 MHz, CDCl_3_) δ 3.86 (s, 3H), 4.04 (s, 2H), 6.90–6.96 (m, 2H), 7.11–7.14 (m, 1H), 7.21–7.36 (m, 6H); ^13^C NMR (75 MHz, CDCl_3_) δ 35.9, 55.4, 110.5, 120.6, 125.8, 127.5, 128.3, 129.0, 129.7, 130.4, 141.1, 157.4; GC–MS (*m*/*z*) 198.2.

**1-Methoxy-2-(pent-4-en-1-yl)benzene (17)** [71]: The title compound **17** was obtained as yellow oil (83 mg, 47% yield) after purification by silica gel column chromatography. ^1^H NMR (300 MHz, CDCl_3_) δ 1.64–1.74 (m, 2H), 2.08–2.15 (m, 2H), 2.61–2.66 (m, 2H), 3.83 (s, 3H), 5.01–5.08 (m, 2H), 5.80–5.94 (m, 1H), 6.84–6.92 (m, 3H), 7.15 (t, *J* = 7.5 Hz, 1H); ^13^C NMR (75 MHz,CDCl_3_) δ 29.2, 33.8, 41.5, 55.4, 110.4, 114.5, 117.9, 120.4, 127.0, 139.1, 145.3, 157.2; GC–MS (*m*/*z*) 176.1.
